# Comprehensive insights into areca nut: active components and omics technologies for bioactivity evaluation and quality control

**DOI:** 10.3389/fphar.2024.1407212

**Published:** 2024-05-30

**Authors:** Yuanyuan Sun, Jian Feng, Wencheng Hou, Huasha Qi, Yangyang Liu

**Affiliations:** ^1^ Key Laboratory of Bioactive Substances and Resources Utilization of Chinese Herbal Medicine, Ministry of Education and National Engineering Laboratory for Breeding of Endangered Medicinal Materials, Institute of Medicinal Plant Development, Chinese Academy of Medical Sciences and Peking Union Medical College, Beijing, China; ^2^ Hainan Provincial Key Laboratory of Resources Conservation and Development of Southern Medicine, International Joint Research Center for Quality of Traditional Chinese Medicine, Hainan Branch of the Institute of Medicinal Plant Development, Chinese Academy of Medical Sciences and Peking Union Medical College, Haikou, China

**Keywords:** areca nut, active components, pharmacology, quality detection, influencing factors, omics, clinical study

## Abstract

Areca nut (AN), the fruit or seed of *Areca catechu* Linn, has many uses, including chewing and medicinal purposes. It has sparked worries about health due to the presence of alkaloids. Chewing AN may have a variety of negative consequences; however, the medicinal use of AN has no notable adverse effects. To completely understand and effectively use AN, researchers have investigated its chemical makeup or biological activity, analyzed the variations between different AN species and different periods, and improved extraction and processing procedures. Today, an increasing number of researchers are exploring the underlying reasons for AN variations, as well as the molecular mechanisms of biosynthesis of chemical components, to comprehend and change AN at the genetic level. This review presents an overview of the clinical study, pharmacology, and detection of the main bioactive components in AN, and the main factors influencing their content, delving into the omics applications in AN research. On the basis of the discussions and summaries, this review identifies current research gaps and proposes future directions for investigation.

## Graphical Abstract

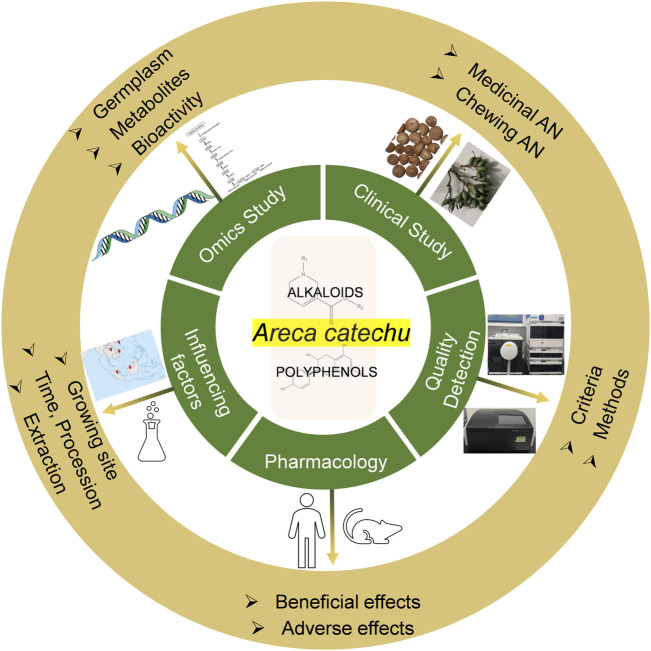

## 1 Introduction

**FIGURE 1:**
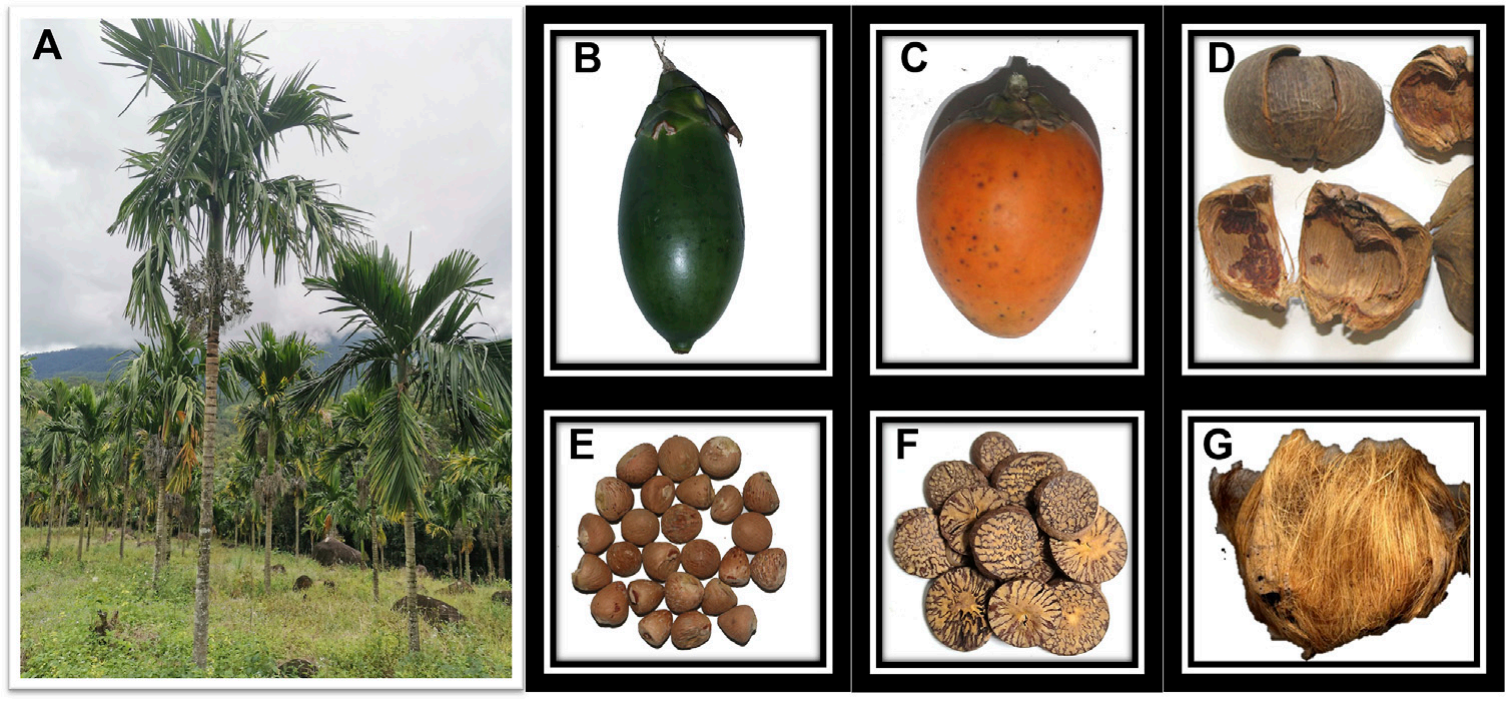
The plant, fruit, husk, and seed of *Areca catechu*. **(A)** Plant *A. catechu*. **(B)** Fresh and tender fruit. **(C)** Ripe fruit. **(D)** Arecae pericarpium. **(E)** Arecae semen. **(F)** Arecae semen tostum. **(G)** Da Fu Mao.

*Areca catechu* is a monoecious, perennial and evergreen tree belonging to the palm family (Figure 1A). It grows in the tropical climates of Asia, East Africa, and the Pacific, particularly in South and Southeast Asian countries. It is now believed that *A. catechu* originated in Indonesia, Malaysia, or the Philippines (Rooney, 1995; Lim, 2012; World Health Organization. Regional Office for the Western Pacific, 2012; Manimekalai et al., 2018; Joo et al., 2020). After thousands of years of evolution, it has become a significant economic tree species in certain regions, such as Indonesia and Vietnam. Hainan Province is the largest producer of *A. catechu* in China (Yang et al., 2018). In addition to its contribution to the agricultural economies of the countries where it is grown, *A. catechu* has an important place in the religious, cultural, and social milieus of the rural people. Areca nut (AN) is the fruit of *A. catechu* (Figures 1B, C), and it is not only used to chew; however, different parts of it have different medicinal values. The immature fruit is boiled and dried, and the husk obtained after removing the seeds is called arecae pericarpium (“Da Fu Pi” in Chinese) (Figure 1D), whereas the husk obtained after processing the mature fruit is called “Da Fu Mao” (Figure 1G). Their primary functions include moving qi, soothing the middle, disinhibiting water, and relieving swelling. The mature fruit is boiled and dried, and the husk is removed, resulting in therapeutic seeds known as arecae semen (Figure 1E). The charred seeds are known as arecae semen tostum (Figure 1F). For seeds, the arecae semen is mainly used to kill parasites, and the arecae semen tostum is mainly used to promote digestion. In this review, the term AN is used to refer to both the fruit and the seed of *A. catechu* without distinction. The fresh young fruit (usually harvested at around 4 months) are chewed with various ingredients in different regions. It is estimated that more than 600 million people chew AN (Warnakulasuriya and Chen, 2022). Unfortunately, AN is considered the world’s fourth most addictive substance and is classified as a Group 1 carcinogen by the International Agency for Research on Cancer (IARC) of the World Health Organization (IARC Working Group on the Evaluation of Carcinogenic Risks to Humans, 2007). In traditional medicinal applications, the processing of traditional Chinese medicine has been proven by numerous studies to enhance efficacy and reduce toxicity. To cater to individuals with diverse constitutions and address various primary symptoms, China has long employed the technique of charcoal roasting to transform arecae semen into arecae semen tostum. Arecae semen has the strongest medicinal properties and is not recommended for frail individuals. By contrast, Arecae semen tostum offers milder effects with less adverse effects while alleviating food stagnation. Researchers have already compared the chemical compositions of AN, in addition to the influence of different processing methods on these chemical components. Distinct parts of AN and various processing techniques can lead to significant changes in the active ingredients.

Although it is true that the medicinal and edible uses of seeds and fruit should not be confused, alkaloids, the main active ingredients that cause the positive effects, also play a major role in the negative effects. As a result, the public is afraid of pharmaceuticals and preparations that contain arecae semen or arecae semen tostum. Nevertheless, the adverse clinical events associated with medicinal seeds are relatively rare and controllable. Ancient books contain no records of arecae semen’s toxicity and usually consider it nontoxic. Only a few records mention likely side effects, such as “fever and injury to vital energy”, emphasizing that it should not be used by those with weak constitutions or those who do not have specific indications for its use (Sun et al., 2017).

This review provides an overview of the clinical study and observation of AN, and two main categories of bioactive compounds in the fruit and seeds of *A. catechu*: alkaloids and polyphenolic substances. It describes their pharmacological effects and potential adverse effects, and summarizes recent literature on the main detected substances, quantification methods, and influencing factors. Subsequently, the application of omics technologies in AN research is discussed, encompassing aspects such as *A. catechu* germplasm, metabolites, and bioactive properties, with the aim of offering a reference for better utilization and quality determination of AN.

## 2 Clinical study of AN

### 2.1 Clinical study of medicinal AN

AN is commonly used in therapeutic settings as a proprietary Chinese medicine. These formulas were proposed by traditional Chinese physicians a hundred or a thousand years ago and were enhanced during use. There have been no reports of clinically significant toxic side effects of the use of AN for medicinal purposes. “Muxiang Binglang Pill” has the effect of eliminating food-induced stagnation, and its combination with Western medicine in the comprehensive treatment of mild acute pancreatitis can effectively promote the recovery of intestinal function and improve its overall clinical efficacy (Wang, 2024). The clinical efficacy of employing “Binglang Powder” in conjunction with acupoint massage in the treatment of patients with spleen and stomach diseases is remarkable, effectively relieving symptoms, reducing inflammatory reactions in the spleen and stomach, and improving spleen and stomach function (Liu, 2021). Moreover, AN can promote the recovery of gastrointestinal function after gynecological abdominal surgery, and its mechanism of action may be related to the modulation of the level of motilin, a brain-gut peptide hormone (Liang et al., 2020). “Food Retention Relieve Oral Liquid” is composed of 10 medicines, including Galli Gigerii Endothelium Corneum, Sparganii Rhizoma, Curcumae Rhizoma, and Arecae semen. It has good clinical efficacy in the treatment of anorexia nervosa in children (Wan, 2010; Sun et al., 2014). “Jinyingzi Binglang Decoction” is effective in treating patients with chronic, intractable diarrhea (Zhang, 2014). “Food Retention and Cough Relieve Oral Liquid for Children” is composed of Crataegi Fructus, Arecae semen, and eight other medicines that are effective in the treatment of pediatric cough and have the advantages of few toxic side effects and ease of use (Di, 2015). “Binglang Shisanwei Pill,” in combination with psychological care, can effectively improve the symptoms of depression and increase satisfaction in clinical care (Gu, 2021). Furthermore, in animal parasite therapy, arecoline hydrobromide is useful in the eradication of cattle tick *Rhipicephalus microplus* infections in calves, and clinical safety has been determined by examining hematological characteristics and performing skin irritation assays in calves infested with ticks (Jain et al., 2021).

### 2.2 Clinical observation of chewing AN

AN chewing often leads to oral problems. Clinical analysis of 189 cases of oral submucosal fibrous degeneration in a hospital in Zhengzhou (a city in China) showed that the onset of oral submucous fibrosis (OSF) predominantly occurs in males, with a tendency for rejuvenation. Age, chewing time, and chewing amount are related to OSF disease severity (Yu et al., 2022). Tang et al. (2018) conducted a clinical analysis of 86 cases of OSF caused by AN chewing; the higher the frequency and duration of AN chewing, the greater the probability of the disease. The majority of patients with OSF have initial symptoms of mucosal irritation pain and limited mouth opening. Most patients also have a smoking habit, which has a synergistic effect on the occurrence of OSF. In oral squamous cell carcinoma (OSCC) patients with an chewing habit, tumors are relatively well differentiated, and the buccal and tongue areas are the most common sites of OSCC (Shao et al., 2021). Long-term AN chewing may lead to different degrees of tooth abrasion. By reviewing the medical records of patients with oropharyngeal squamous cell carcinoma (OPSCC) between 1999 and 2013, Chen et al. (2020) found that HPV-OPSCC accounted for the majority of OPSCC in areas where AN chewing is endemic. The combination of alcohol/betel nut chewing/smoking exposure has a significant negative impact on disease-free survival and overall survival.

## 3 Alkaloid

Alkaloids display a wide range of biological functions and may pose potential health threats. They are typical constituents and bioactive compounds found in AN, with presence primarily limited to *A. catechu* in the palm family. Presently, at least 18 alkaloids have been identified in AN (Table 1) (Peng et al., 2015; Tang et al., 2017; Cao et al., 2019); however, there is a notable dearth of dedicated research on the isolation and identification of alkaloids in the seed. The total alkaloid content in AN varies between 0.3% and 0.6% (Han, 2010). Arecoline is commonly considered the most abundant alkaloid, followed by guvacine, arecaidine, and guvacoline. These four alkaloids not only constitute a significant portion of the alkaloids in AN but are also major active substances (Oliveira et al., 2021). Hence, researchers often rely on measuring the content of arecoline or these four alkaloids as evidence of AN efficacy or toxicity. Furthermore, experiments have shown that different regions, growth stages, and processing methods can significantly influence the alkaloid content of AN.

**TABLE 1:** Chemical compounds isolated from AN.

Classification	No.	Chemical component	References
Alkaloids	1	Arecoline	*
	2	Arecaidine	*
	3	Guavacoline	*
	4	Guavacine	*
	5	Arecolidine	*
	6	Ethyl N-methyl-1,2,5,6-tetrahydro-pyridine-3-carboxylate	*
	7	Methyl nicotinate	*
	8	Ethyl nicotinate	*
	9	Methyl N-methylpiperidine-3-carboxylate	*
	10	Ethyl Nmethylpiperidine-3-carboxylate	*
	11	Nicotine	*
	12	Isoguvacine	*
	13	Homoarecoline	*
	14	Arecatemines A	*
	15	Arecatemines B	*
	16	Arecatemines C	*
	17	Acatechu A	*
	18	Acatechu B	*
Polyphenol	19	Isorhamnetin	*
	20	Chrysoeriol	*
	21	Luteolin	*
	22	Quercetin	*
	23	4′, 5′-dihydroxy3′, 5′, 7′-trimethoxyflavonone	*
	24	5, 7, 4′-trihydroxy-3′, 5′dimethoxy flavanone	*
	25	Liquiritigenin	*
	26	Jacareubin	*
	27	Catechins	*
	28	Epicatechins	*
	29	Procyanidin A1	*
	30	Procyanidin B1	*
	31	Procyanidin B2	*
	32	Arecatannin A1	*
	33	Arecatannin B1	*
	34	Arecatannin C1	*
	35	Arecatannin A2	*
	36	Arecatannin A3	*
	37	Arecatannin B2	*
	38	Tannic acid	Yin et al. (2021)
	39	Gallic acid	Wang et al. (2011)
	40	Epigallocatechin gallate	Wang et al. (2011)
	41	Epicatechin-(4β→8)-epicatechin (4β→8)-catechin	Mu et al. (2014)
	42	Epicatechin-(4β→6)-epicatechin-(4β→8)-catechin	Mu et al. (2014)
	43	Procyanidinb7	Mu et al. (2014)
	44	Femenol	Peng (2018)
	45	Vanillic acid	Yang et al. (2012)
	46	Trans-3,4′,5-trihydroxistilbene	Yang et al. (2012)
	47	Resveratrol	Yang et al. (2012)
	48	Ferulic acid	Yang et al. (2012)
Triterpenes	49	Pelargonidin chloride	Peng (2018)
	50	Ursonic acid	*
	51	3β-acetyl ursolic acid	*
	52	Aromatic alcohol	*
	53	Arborinol methyl ether	*
	54	Fernenol	*
	55	Arborinol	*
Steroids	56	Arundoin	*
	57	Β-sitosterol	*
	58	Cycloartenol	Yang et al. (2012)
	59	Stigmata-4-en-3-one	*
	60	5,8-epidioxiergosta-6,22-dien-3β-ol	*
Fatty acids	61	De-O-methyllasiodiplodin	Yang et al. (2012)
	62	Lauric acid	*
	63	Myristic acid	*
	64	Palmitic acid	*
	65	Stearic acid	*
	66	Oleic acid	*
	67	Capric acid	Zeng (2012)
	68	Octanoic acid	Zeng (2012)
	69	Linoleic acid	Zeng (2012)
	70	Heptadecanoic acid	Zeng (2012)
	71	Dodecenoic acid	Shen and Duan (2009)
	72	Tetradecenic acid	Shen and Duan (2009)
	73	2-Hexadecenoic acid	Guo et al. (2012)
	74	Nonanoic acid	Guo et al. (2012)
	75	Benzoic acid	Guo et al. (2012)
	76	Pentadecanoic acid	Guo et al. (2012)
other compounds	77	Chrysophanol	*
	78	Physcion	*
	79	P-hydroxybenzoic acid	*
	80	Epoxyconiferyl alcohol	*
	81	4-[30-(hydroxymethyl)oxiran-20-yl]2,6-dimethoxyphenol	*
	82	Protocatechuic acid	*
	83	Isovanillic acid	*
	84	Cyclo-(Leu-Tyr)	*
	85	Quinic acid	Liu and Chang (2023)
	86	Vanillin	Liu and Chang (2023)
	87	Alphaterpineol	Liu and Chang (2023)
	88	Benzyl alcohol	Liu and Chang (2023)

Some of the content in this table is quoted from Peng et al. (2015), and the references are indicated by the symbol *.

### 3.1 Pharmacological activity and alkaloid toxicity in AN

#### 3.1.1 Beneficial effects of alkaloids in AN

The alkaloids in AN exhibit a wide range of biological effects, including anthelmintic, antimicrobial, anti-inflammatory, and analgesic effects and effects on the cardiovascular, nervous, digestive, and endocrine systems.

##### 3.1.1.1 Anthelmintic effect

The Chinese Pharmacopoeia documents that arecae semen can be used for anthelmintic purposes (National Pharmacopoeia Committee, 2020). Alkaloids present in AN have exhibited significant anthelminthic effects against various endoparasites in the body, such as hydatid worms, tapeworms, and acute *Toxoplasma gondii* parasites (Batham, 1946; Zheng et al., 1998; Xu, 2018). In a study conducted by Puyathorn et al. (2022), the methanol and water crude extracts from AN, as well as the arecoline hydrobromide solution, were capable of damaging the tegument (surface membrane) of flukes and effectively eliminating them in the early stages.

##### 3.1.1.2 Antimicrobial effect

The alkaloids in AN also display inhibitory effects on *Proteusbacillus vulgaris*, *Candida albicans*, and *Bacillus anthracis* spores, with a minimal inhibitory concentration (MIC) of 0.8 mg/mL (Luo et al., 2010). The alkaloids and polyphenolic compounds in AN extracts can also effectively control the occurrence of anthracnose diseases (Ahmad Rusdan et al., 2015).

##### 3.1.1.3 Hypotensive effect

AN primarily contains arecoline and condensed tannins, and these components have a relaxing effect on the aortas of rats with an intact endothelium. Thus, arecoline can reduce vascular tone in a concentration-dependent manner, triggering a blood pressure-lowering effect (Goto et al., 1997; Chen et al., 2020).

##### 3.1.1.4 Anti-inflammatory activity

Arecoline is an agonist of the α7 nicotinic acetylcholine receptor and an effective inflammation inhibitor. Papke et al. (2015) found that when used in combination with the positive allosteric modulator PNU120696, arecoline and PNU120696 together significantly upregulate the expression of this receptor, thereby achieving a synergistic anti-inflammatory effect.

##### 3.1.1.5 Effects on the nervous system

Arecoline is the primary substance responsible for the effects on the central and autonomic nervous systems. Arecoline has a stimulating effect on the sympathetic nervous system and can simultaneously target M and N receptors. Alkaloids found in AN, including arecoline, arecaidine, guvacine, and guvacoline, can induce behavioral changes in zebrafish (Siregar et al., 2022). These changes include an increase in the sense of wellbeing, stamina, and euphoria (Garg et al., 2014). Research suggests that the injection of arecoline significantly shortens sleep duration in mice exposed to alcohol but prolongs their resistance to sleep following pentobarbital injection (Sun et al., 2005; Xiao et al., 2013). Nevertheless, experiments targeting the righting reflex induced by pentobarbital and ethanol have shown that arecoline can reduce the sleep duration induced by ethanol but does not affect the sleep duration induced by pentobarbital at the doses used. Additionally, at the same dosage levels, arecoline does not significantly affect the latency of sleep induced by ethanol or pentobarbital in mice (Sun et al., 2010). When administered in the early stages of aging, arecoline exhibits potential pharmacological properties for extending lifespan. Studies suggest that arecoline reduces muscle tissue through the GAR2/PLCβ pathway, leading to lifespan extension (Ching et al., 2020). Furthermore, doses of 125 and 175 mg/kg of the dichloromethane extract of AN significantly inhibit withdrawal symptoms in morphine-dependent mice. Arecoline also improves some of the negative symptoms of mental illness (Kumarnsit et al., 2005). In zebrafish, arecoline increases serotonin levels, social preference, and brain norepinephrine while reducing serotonin turnover, leading to anxiolytic effects. Thus, it can be seen that arecoline possesses anxiolytic-like activity (Serikuly et al., 2021). Arecoline enhances cognition, memory, and some behavioral disorders in patients with schizophrenia or Alzheimer’s disease by activating postsynaptic muscarinic M1 receptors (Avery et al., 1997; Brunetti et al., 2020).

##### 3.1.1.6 Effects on the digestive system

Previous research has reported that a water extract containing 0.06% arecoline significantly enhances the contraction of gastric smooth muscle and muscle strips in the duodenum, ileum, and colon (Ni et al., 2004). Wang et al. (2020) found that hydrobromide arecoline noticeably increased the contractile ability of gastric and intestinal muscle strips in various regions of the gastrointestinal tract in wild-type C57BL/6 mice. Correspondingly, Arecae pericarpium extract and arecoline can induce contractions in the lower esophageal sphincter sling and clasp muscles in pigs, and this effect is dose-dependent. The contractions induced by the extract and arecoline are mediated by muscarinic receptors, suggesting the possibility of developing alternative treatments for gastroesophageal reflux disease (Tey et al., 2021).

##### 3.1.1.7 Effects on the endocrine system

AN has an impact on the human endocrine system. Arecoline can increase the release of corticotropin-releasing hormones by activating the hypothalamic–pituitary–adrenal axis (Calogero et al., 1989; Wang and Hu, 2010). Dasgupta’s team found that arecoline can exacerbate thyroid dysfunction in mice under metabolic stress, while also improving thyroid hyperactivity induced by cold stress (Dasgupta et al., 2017; Dasgupta et al., 2018). Wang et al. (2008) discovered that by activating L-type calcium channels, arecoline increases the activity of 17β-hydroxysteroid dehydrogenase and enhances the expression of Steroidogenic Acute Regulatory Protein in interstitial cells of the testes. This directly stimulates the production of testicular hormones. Qi (2010) found that arecoline may activate insulin receptor substrates in the insulin signaling pathway through the phosphatidylinositol 3-kinase/protein kinase B (PI3K/AKT) pathway, which promotes insulin secretion to lower blood glucose levels.

##### 3.1.1.8 Anticancer activity

Arecoline can reduce the levels of the epithelial tumor cell survival factor IL-6, increase the levels of tumor suppressor P53, induce cell cycle arrest, promote apoptosis, and thereby prevent basal cell carcinoma (Huang et al., 2012).

#### 3.1.2 Adverse effects of alkaloids in AN

Nevertheless, the higher alkaloid content in the seed than in the husk makes the seed the primary carcinogenic component, especially in oral cancer development. *In vitro* and *in vivo* experiments have shown that arecoline can induce the generation of reactive oxygen species, cell cycle arrest, apoptosis, DNA damage, upregulation of transcriptional proteins, and cytotoxicity in human endothelial cells (Tseng et al., 2012; Peng et al., 2015; Wang et al., 2016; Tu et al., 2019).

##### 3.1.2.1 Fibrosis

It has been reported that four alkaloids, namely, arecoline (the most potent), arecaidine, guvacine, and guvacoline, stimulate fibroblasts to produce collagen, which is believed to represent the main pathogenic mechanism of oral submucous fibrosis (Angadi and Rao, 2011; Arakeri and Brennan, 2013). Among these, arecoline, the key compound initiating the process of submucous fibrosis in the oral mucosa, operates in conjunction with Transforming Growth Factor-β, which plays a pathological role in organ fibrosis (Li et al., 2019; Ku et al., 2021). Arecoline promotes the activation of buccal mucosal fibroblasts, which is induced by transforming growth factor-β1 through the enhancement of phosphodiesterase 4A activity (Zhang et al., 2021). Additionally, arecoline causes cardiac fibrosis, regulates renal fibrosis in renal tubular epithelial cells, and ultimately contributes to the progression of chronic kidney disease (Ding et al., 2018; Hsieh et al., 2020; Ku et al., 2021).

##### 3.1.2.2 Cancer cell metastasis

Arecoline has been shown to enhance cervical lymph node metastasis in tongue xenografted models using nude mice, indicating that it induces the epithelial–mesenchymal transition and promotes metastasis in oral cancer (Ren et al., 2021). Additionally, arecoline has demonstrated the ability to stimulate proliferation and enhance the migratory potential of HepG2 cells, upregulating the expression of PI3K-AKT pathway factors. This suggests that arecoline can promote the migration and proliferation of human hepatocellular carcinoma cells by activating the phosphatidylinositol 3-kinase/protein kinase B/mammalian target of the rapamycin (PI3K/AKT/mTOR) pathway (Xie et al., 2022). However, there is no direct evidence of arecoline-induced carcinogenesis in animal models (Shen et al., 2020).

##### 3.1.2.3 Cytotoxicity

Arecoline stimulates collagen production in cultured cells at concentrations as low as 0.1 μg/mL and is cytotoxic at concentrations above 10 μg/mL (Cox et al., 2010). It suppresses epithelial cell viability through the AKT/mTOR signaling pathway in oral cancer, with minimum effective concentrations ranging from 0.025 to 12.5 μg/mL (Chen et al., 2014; Gu et al., 2019). AN’s ethanolic extract exhibited cytotoxicity in a lung cancer cell line at a concentration of 1.7 µg arecoline per mg of extract (Abbas et al., 2018). In a breast cancer cell line study, arecoline inhibited cell proliferation and induced apoptosis at a concentration of 100 μM/L (Feng et al., 2016). Arecoline reduces the aminobutyric acid functional pathway in the nervous system and induces neuronal damage or neuronal apoptotic death by attenuating antioxidant defense and enhancing oxidative stress (Huang et al., 2003; Shih et al., 2010). When intraperitoneally injected into rats, arecoline induces cardiac apoptosis through the Fas/Fas ligand pathway (Lin et al., 2021). Additionally, different doses of arecoline exhibit varying degrees of impact on murine splenic lymphocytes, human liver cells, C2C12 myoblasts, and other cell types (Chou et al., 2008; Dasgupta et al., 2010; Chang et al., 2012; Chang et al., 2013; Gu et al., 2013). These findings collectively indicate that arecoline has cytotoxic and apoptotic effects on various cell types and can impact different signaling pathways and biological processes. These effects can vary depending on the cell type and concentration of arecoline used. Arecoline is a major alkaloid found in AN, and its health effects are of concern due to its association with oral cancers and other health problems in individuals who consume AN.

##### 3.1.2.4 Reproductive toxicity

In recent years, comprehensive research has been conducted on the toxic effects of AN and arecoline on reproduction. Garcia-Algar et al. (2005) reported the birth outcomes of newborns whose mothers consumed AN during pregnancy, with two exhibiting adverse outcomes; however, no clear conclusions can be drawn regarding the correlation between fetal exposure to arecoline and clinical outcomes. Moreover, an experiment conducted by Liu et al. (2011) demonstrated that arecoline is toxic to embryos during the peri-implantation stages in mice. Additionally, alkaloids significantly decrease the motility of human sperm in a dose-dependent manner (Yuan et al., 2012). Another study showed that a dose of 50 mg/kg body weight of AN has contraceptive effects in male rats (Rahman et al., 2020).

##### 3.1.2.5 Genotoxicity

Arecoline, in particular, is one of the genotoxic alkaloids in AN. Arecoline could inhibit p53 by its expression and transactivation function. Consequently, this inhibits DNA repair and induces the DNA damage response (Tsai et al., 2008). In addition, it may trigger DNA chain disruption, chromosome distortion, and sister chromatid exchange, affect DNA repair, and cause oxidative stress (IARC Monographs Vol 128 group., 2021).

##### 3.1.2.6 Other effects

**FIGURE 2:**
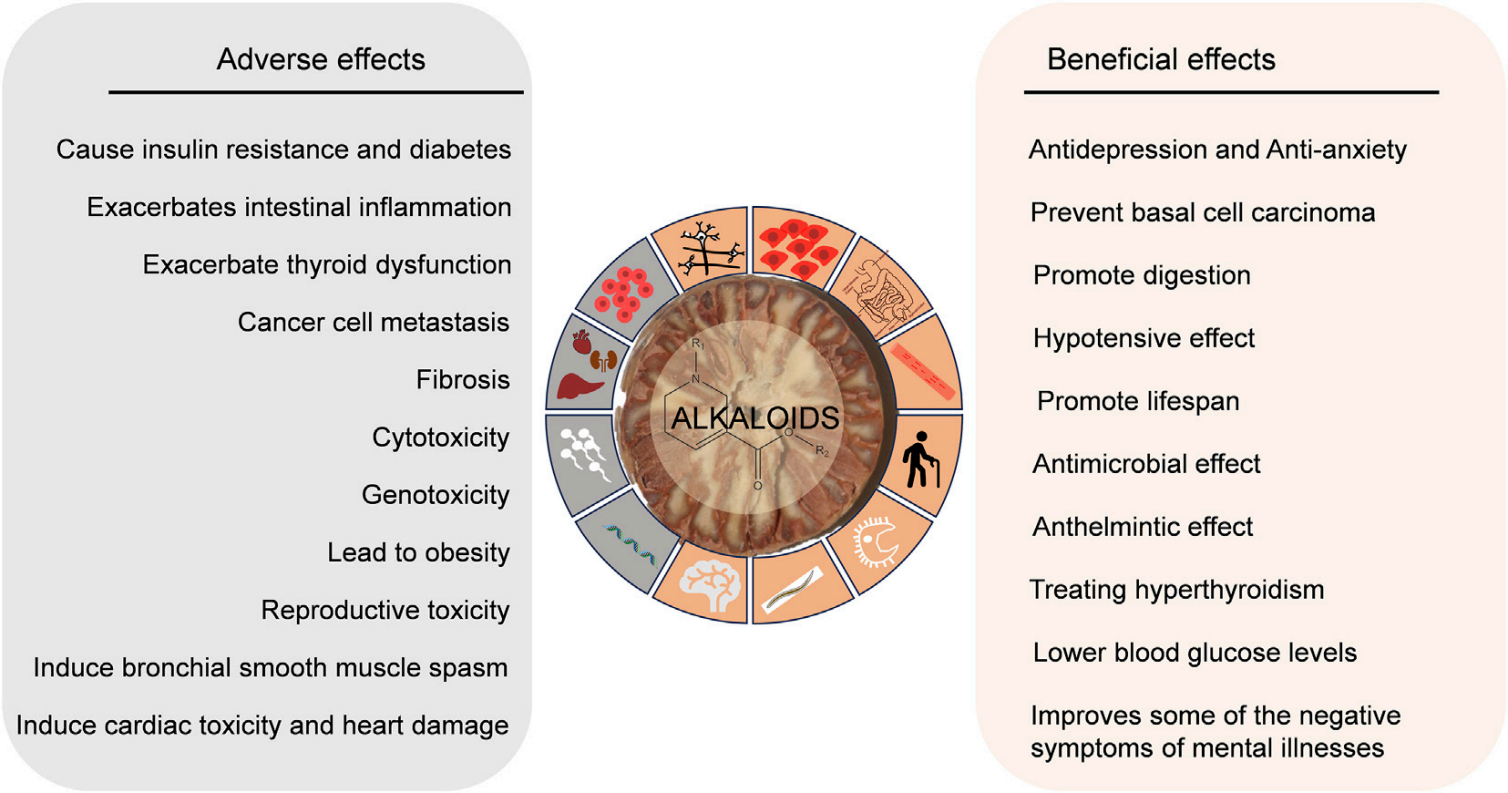
The beneficial and adverse effects of alkaloids in AN.

In addition, arecoline can induce cardiac toxicity and heart damage through various pathways, including the activation of JAK2/STAT3 induced by IL-6, MEK5/ERK5, and Mitogen-Activated Protein Kinase (Ho et al., 2022). Arecoline disrupts mouse oocytes’ low adenosine 5′-triphosphate levels, increases oxidative stress, and exacerbates intestinal inflammation by affecting the abundance of gut microbiota, which regulates serum metabolite concentrations in mice (Li et al., 2020; Zhao et al., 2023). In addition, arecoline may induce bronchial smooth muscle contraction and spasm, causing chest tightness; affect lipid metabolism thus leading to obesity; and may also cause insulin resistance and diabetes mellitus (Taylor et al., 1992; Hsu et al., 2010; Hsieh et al., 2011). The beneficial and adverse effects of AN are shown in Table 2 and Figure 2.

**TABLE 2:** The pharmacological activities and toxicity of alkaloids in AN.

Diseases/tissues	Compounds		Bioactivities	Text model	References
Beneficial effects					
Endoparasites		Arecoline and arecoline hydrobromide	Eliminate acute *Toxoplasma gondii* parasites	Mice	Xu (2018)
		Arecoline hydrobromide	Kill flukes	Rumen fluke	Puyathorn et al. (2022)
		Arecoline and arecoline hydrobromide	Against tapeworms, hydatid worms	Dog	Batham (1946)
Microbial infection		Alkaloids	Control the occurrence of anthracnose disease	Mangoes	Ahmad Rusdan et al. (2015)
		Arecoline	Against *Proteusbacillus vulgaris*, *Candida albicans* and *Bacillus anthracis*	*Escherichia coli*, *Staphylococcus aureus*, *Proteusbacillus vulgaris*, *Bacillus sublilis*, *Candida albicans*, *Bacillus anthracis*, *Aspergillus niger*, *Penicillium*	Luo et al. (2010)
Blood pressure		Arecoline	Reduce vascular tone	Rats	Chen et al. (2020b)
		Arecoline and condensed tannin	Relaxes the rat aorta	Rats	Goto et al. (1997)
Inflammation		Arecoline	Anti-inflammatory effect	*Xenopus* oocytes	Papke et al. (2015)
Nervous system		Arecoline, arecaidine, guvacine, and guvacoline	Increase in the sense of wellbeing, stamina, and euphoria	Zebrafish	Siregar et al. (2022)
		Arecoline	Shortens sleep duration in mice exposed to alcohol while prolonging their resistance to sleep following pentobarbital injection	Mice	Sun et al. (2005), Xiao et al. (2013)
		Arecoline	Arecoline can reduce the sleep duration induced by ethanol but does not affect the sleep duration induced by pentobarbital at the doses used	Mice	Sun et al. (2010)
		Arecoline	Promote lifespan	*Caenorhabditis elegans*	Ching et al. (2020)
		Arecoline	Improves some of the negative symptoms of mental illnesses	Mice	Kumarnsit et al. (2005)
		Arecoline	Anxiolytic-like activity	Zebrafish	Serikuly et al. (2021)
		Arecoline	Enhances cognition, memory, and some behavioral disorders in patients with schizophrenia or Alzheimer’s disease	Human	Avery et al. (1997), Brunetti et al. (2020)
Digestive system		Water extract containing 0.06% arecoline	Enhances the contraction of gastric smooth muscle and muscle strips in the duodenum, ileum, and colon	Human vaginal/buccal *in vitro* model	Ni et al. (2004)
		Hydrobromide arecoline	Increases the contractile ability of gastric and intestinal muscle strips in various regions of the gastrointestinal tract	C57BL/6 and W/Wv Mutant Mice	Wang et al. (2020)
		Areca pericarpium extract and arecoline	Induce contractions in the lower esophageal sphincter sling and clasp muscles	Pigs	Tey et al. (2021)
Endocrine system		Arecoline	Increase the release of corticotropin-releasing hormone	Male Sprague-Dawley rats, anterior pituicytes and adrenocortical cells	Calogero et al. (1989)
		Arecoline	Treating hyperthyroidism	Mice	Dasgupta et al. (2018)
		Arecoline	Stimulates the production of testicular hormones	Rats	Wang et al. (2008)
		Arecoline	Lower blood glucose levels	Rats	Qi (2010)
Cancer		Arecoline	Prevent basal cell carcinoma	Human basal cell carcinoma cells	Huang et al. (2012)
Adverse effects					
Cancer	Arecoline		Induces epithelial-mesenchymal transition and promotes metastasis in oral cancer	Oral squamous cell carcinoma cells	Ren et al. (2021)
	Arecoline		Promote the migration and proliferation of human hepatocellular carcinoma cells	Human hepg2 cells	Xie et al. (2022)
**Fibrosis**	Arecoline, arecaidine, guvacine, and guvacoline		Oral submucous fibrosis	BALB/c mice, HUVEC cells; rats	Li et al. (2019), Ku et al. (2021)
	Arecoline		Promotes the activation of buccal mucosal fibroblasts	Buccal mucosal fibroblast	Zhang et al. (2021)
	Arecoline		Cardiac fibrosis	Rats	Ku et al. (2021)
	Arecoline		Renal fibrosis	Tubular epithelial cells	Ding et al. (2018)
	Arecoline		Chronic kidney disease	Human kidney cells	Hsieh et al. (2020)
**Cytotoxicity**	Arecoline		Cytotoxic at concentrations above 10 μg/mL	Human	Cox et al. (2010)
	Arecoline		Suppressed epithelial cell viability (from 0.025 μg/mL to 12.5 μg/mL)	Normal human gingival fibroblasts and Ca9-22 cells; epithelial cell	Chen et al. (2014), Gu et al. (2019)
	Arecoline		Cytotoxicity	Lung cancer cell line	Abbas et al. (2018)
	Arecoline		Inhibited cell proliferation and induced apoptosis at a concentration of 100 μM/L	MCF-7 human breast cancer cells	Feng et al. (2016)
	Arecoline		Induces neuronal apoptotic death within a concentration range of 50–200 µM	Rat primary cortical neurons cells	Shih et al. (2010)
	Arecoline		Reduces the aminobutyric acid functional pathway in the nervous system and induces neuronal damage	Human	Huang et al. (2003)
	Arecoline		Induced cardiac apoptosis	Sprague–Dawley rat	Lin et al. (2021)
	Arecoline and aqueous extract of AN		Impact on murine hepatocyte	Mice	Gu et al. (2013)
	Arecoline		Growth arrest	Rat hepatocytes	Chou et al. (2008)
	Arecoline		Detrimental effect to muscle development	C2C12 myoblastic cells	Chang et al. (2012), Chang et al. (2013)
**Reproductive toxicity**	Arecoline		Embryotoxicity	Mice	Liu et al. (2011)
	Arecoline		Ecrease the motility of human sperm	Human sperm	Yuan et al. (2012)
	Arecoline		Contraceptive effects	Male rats	Rahman et al. (2020)
**Genotoxicity**	Arecoline, Arecaidine, Arecoline-N-oxide		Induce DNA chain disruption, chromosome distortion, and sister chromatid exchange, affect DNA repair, and cause oxidative stress	Human	IARC Monographs Vol 128 group. (2021)
	Arecoline		Inhibits p53, represses DNA repair, and triggers DNA damage response	KB cells, hep-2 cells, 293 and H1299 cells	Tsai et al. (2008)
Other	Arecoline		Induce cardiac toxicity and heart damage	Sprague–Dawley rats	Ho et al. (2022)
	Arecoline		Exacerbates intestinal inflammation	Mouse oocyte; mice	Li et al. (2020), Zhao et al. (2023)
	Arecoline		Exacerbate thyroid dysfunction in mice under metabolic stress	Mice	Dasgupta et al. (2017)
	Arecoline		May result in insulin resistance and diabetes	3T3-L1 preadipocytes	Hsieh et al. (2011)
	Arecoline		Induces bronchial smooth muscle contraction and spasm, causing chest tightness	Bangladeshis asthmatic patients, human	Taylor et al. (1992)
	Arecoline		Affect lipid metabolism and lead to obesity	Human	Hsu et al. (2010)

### 3.2 Alkaloid detection criteria and methods in AN

Researchers typically evaluate the effectiveness and safety of AN by measuring the alkaloid content. There is a thorough summary of the methodologies for liquid chromatography (LC) separation of alkaloids in herbal medicines provided by Zhao et al. (2020), including reversed-phase LC, ion exchange chromatography, hydrophilic interaction chromatography, two-dimensional LC, and mixed-mode LC. High-performance liquid chromatography (HPLC) is the primary method used for quantitative analysis in AN.

Xue et al. (2011) established a near-infrared spectroscopy model to predict the dynamic changes in the content of three alkaloids (arecoline, arecaidine, and guvacine) during the drying process of AN. This model reveals the quality changes in AN during the drying process, providing a basis for rapid online analysis and quality control. Yuan et al. (2012) improved HPLC by employing a cation exchange column to determine the content of three major alkaloids, arecoline, arecaidine, and guvacine, in fresh, dried, shell, and nut parts to investigate the impact of AN on male sperm. Tian et al. (2018) used HPLC to determine the content of four alkaloid components (arecoline, arecaidine, guvacine, and guvacoline) in AN materials. Their findings indicated that arecoline, arecaidine, guvacine, and guvacoline could serve as quality control markers for AN materials, with specific recommendations for the contents in various parts of the nut. Yuan et al. (2018) reported the use of both HPLC-UV and ultra-high-performance liquid chromatography-tandem mass spectrometry (UHPLC-MS/MS) methods for the quantitative analysis of four characteristic AN alkaloids. They introduced an UHPLC-MS/MS method to assess typically overlooked components in AN products, including eight phenolic compounds and a trace number of alkaloids. Gheddar et al. (2020) developed two methods for analyzing four alkaloids in hair to assess long-term AN exposure. The presence of these substances in hair serves as a reliable marker of AN consumption. Hu et al. (2010) conducted a high-throughput analysis of five urinary metabolites of AN and tobacco alkaloids using online solid-phase extraction liquid chromatography–tandem mass spectrometry to aid in the study of the carcinogenic effects of AN and tobacco exposure. The detected urinary metabolites of AN included arecoline, arecaidine, and N-methylnipecotic acid. Osborne et al. (2022), to determine the levels of five alkaloids as markers to evaluate the safety of chewing AN, used UHPLC-MS/MS. Huang (2022) used HPLC to measure four cytotoxic alkaloids in AN. Pasupuleti et al. (2022) presented a green analytical methodology combining in-syringe-assisted vortex-induced salt-enhanced liquid-liquid microextraction with UHPLC-MS/MS to simultaneously monitor five AN alkaloids (arecaidine, guvacine, guvacoline, arecoline, and N-Nitrosoguvacoline) in saliva and urine, aiming to predict the potential health risks of short- and long-term betel quid chewing. Liu et al. (2022) established a liquid chromatography-ion trap-time of flight mass spectrometry method for the simultaneous determination of four areca-specific alkaloids and the analysis of the two main toxic alkaloids (arecoline and arecaidine) during AN blanching. Additionally, a combination technique of HPLC coupled with ion mobility spectrometry (IMS) was established by Lu et al. (2022) to detect the contents of arecoline, guvacine, and arecaidine in husks, seeds, green fruit, smoked fruit, and simotang oral liquid. Wu et al. (2022) used matrix-assisted laser desorption/ionization mass imaging spectrometry (MALDI-MSI) to detect 10 physiologically active alkaloids in different AN fruit developmental stages, with LC-MS/MS validation and quantification to assess the distribution of these alkaloids in various tissue types. Furthermore, given that the detection of arecoline relies primarily on time-consuming and expensive chromatography-based methods, Wang et al. (2022) developed an indirect competitive enzyme-linked immunosorbent assay (icELISA) method using mAb-A5H12 for the detection of arecoline in traditional Chinese medicine and fresh AN. Detailed information is summarized in Table 3.

**TABLE 3:** Detection of alkaloids.

Test substance	Aim	Test method	Instrument	Condition	References
Arecoline, arecaidine, and guvacine	Reflect the quality changes in AN during the drying process	Bruker Matrix-I FT-NIR spectrometer; HPLC: Agilent 1,100 Series HPLC system with UV–Vis detector	Nucleosil 100–5 SA column, 250 × 4.6 mm, 5 μm	Methanol: water: phosphoric acid (60:40:0.3, pH 3.8, adjusted by 25% of ammonia); 212 nm	Xue et al. (2011)
Arecoline, arecaidine, and guvacine	Investigate the impact of alkaloids in fresh, dried, shell, and nut parts of AN on male sperm	HPLC: Agilent 1,100 Series HPLC system	Whatman Partisil SCX, 4.6 mm × 250 mm, 10 μm; 1.0 mL/min; 25°C	Acetonitrile: 0.5% orthophosphoric acid (pH 3.8 with triethanolamine) 60:40; 215 nm	Yuan et al. (2012)
Arecoline, arecaidine, guvacine, and guvacoline	Determine the content of four alkaloid components in various parts of the nut to serve as quality control markers for AN materials	HPLC: Prominence LC-20A HPLC system with SPD-M20 A Diode array detector	Welch SCX, 4.6 mm × 250 mm, 5μm; 0.5 mL/min; 35°C	Acetonitrile: 0.2% aqueous phosphoric acid solution (pH 3.85–3.90 with concentrated ammonia) 50:50; 215 nm	Tian et al. (2018)
Arecaidine, guvacine, guvacoline, arecolin (HPLC-UV); Protocatechuate, (+)-Catechin, Methyl nicotinate, p-Hydroxybenzoic acid, Vanillic acid, (+)-Epicatechin, (+)-Liquiritigenin, Luteolin, Isorhamnetin (UHPLC-MS/MS)	Reveal substantial variations in the levels of areca alkaloids among Qingguo, Yanguo, raw husk, and the seed products for comprehensive quality control of Binglang products	HPLC-UV: Agilent 1,260 Infinity system equipped with diode array detector; UHPLC-MS/MS: Agilent 1,200 Infinity Series UHPLC instrument coupled with an Agilent 6,460 Triple Quadrupole tandem mass spectrometer	WTpolysulfonix-SCX column, 4.6 × 250 mm, 5μm; 1.0 mL/min; 30°C. 1.8 mm Agilent Elipse C18 (2.1 × 50 mm); 0.3 mL/min; 30°C	Acetonitrile: 0.1% phosphoric acid (pH 3.0 adjusted by concentrated ammonia solution) 81:19; 215 nm. 0.1% formic acid in water (A) and methanol (B). The linear gradient was as follows: 0–5 min, 20% B; 5–6 min, 20%–60% B; 6–9 min, 60%–90% B; 9–20 min, 90% B	Yuan et al. (2018)
Arecaidine, guvacine, guvacoline, arecolin	Analyze four alkaloids in hair to serve as a reliable marker for AN consumption	UPLC-MS/MS: Waters Acquity UPLCTM system; UPLC-Q-Tof-MS: XEVOTM G2XS Q-TOF mass spectrometer	Acquity UPLC HSS C18 column, 150 mm × 2.1 mm, 1.8μm; 0.3 mL/min; 50°C	Water-0.1% formic acid (A) and MeOH-0.1% formic acid (B), A: 0 min, 98%; 4.00 min, 98%; 4.20 min, 5%; 4.60 min, 5%; 4.70 min, 98%; and 8.00 min, 98%	Gheddar et al. (2020)
Arecoline, arecaidine, and N-methylnipecotic acid	Study the carcinogenic effects of AN and tobacco exposure	Online solid-phase extraction liquid chromatography-tandem mass spectrometry: Agilent 1,100 series autosampler and binary pump	ODS-3 C^18^ trap column, 75 × 2.1 mm, 5 μm; 200 μL/min	2% methanol: 0.1% TFA	Hu et al. (2010)
Arecoline, arecaidine, guvacoline, guvacine, N-Nitroso guvacoline	Determine the levels of five alkaloids as markers to evaluate the safety of chewing AN	UHPLC-MS/MS: UHPLC with autosampler Shimadzu Nexera-I 2040C 3D system, LCMS-8045 mass spectrometer equipped with an electrospray-ionization source	C18-PFP column, 150 × 4.6 mm, 3 μm; 400 μL/min	0.05% FA (A) in pure water and mobile phase methanol (B)	Osborne et al. (2022)
Arecoline, arecaidine, guvacoline, guvacine	Determine four kinds of cytotoxic alkaloids in Areca Semen for the quality control	HPLC: Waters e2695	Agilent ZORBAX C18, 250 mm × 4.6 mm, 5μm; 1.0 mL/min; 30°C	Methanol: 0.05 mol/L diammonium hydrogen phosphate solution (pH 6.8–7.0) 63:37; 215 nm	Huang (2022)
Arecoline, arecaidine, guvacoline, guvacine, N-Nitroso guvacoline	Biomonitoring five toxic areca nut alkaloids for predicting health hazardous risks	UHPLC-MS/MS: Shimadzu i-series Ultra HPLC system equipped with an autosampler, Shimadzu triple-quadrupole LCMS-8045 by electrospray-ionization (ESI)	ACE C18-PFP HPLC column, 150 × 4.6 mm, 3 µm; 0.40 mL/min	0.05% FA in water (A) and methanol (B); 0–0.5 min increased from 25% (B) to 60% (B); 0.5–4.0 min raised from 60% (B) to 90% (B) hold at 90% (B) for 8 min; 12.0–15 min decreased from 90% (B) to 25% (B) re-equilibrate for 5.0 min	Pasupuleti et al. (2022)
Arecoline, arecaidine, guvacoline, and guvacine	Estimate variations in areca alkaloids levels during areca processing	LCMS-IT-TOF: LC-30 AD binary pump, SIL-20 AC autosampler, CTO-20 AC column oven, and SPD-M20A DAD.	ACQUITY UPLC^®^ BEH C18 column, 100 mm × 2.1 mm, 1.7 μm; 0.3 mL/min; 40°C	Water containing 10 mmol/L NH4OAc (A) and methanol containing 10 mmol/L NH4OAc (B); 0–13.00 min, 10%–70% (B), 13.00–16.00 min 70% (B); 16.00–16.10 min, 70%–10% (B), 16.10–20.10 min 10% (B); 10 μl; 215 nm	Liu et al. (2022)
Arecoline, guvacine, and arecaidine	Quantitative determination of major alkaloids in areca nut products	High-performance liquid chromatography coupled with ion mobility spectrometry: Wufeng LC100 HPLC instrument with a UV detector	X-Bridge reversed-phase C18 column, 4.6 × 250 mm, 5 μm; 0.4 mL/min	water with 0.5% ammonia (A) and methanol (B); A linear gradient elution program of 45%–80% B from 0 to 30 min; 215 nm	Lu et al. (2022)
Arecoline, arecaidine, caffeine, cotinine, guvacine, guvacoline, hordenine, sophoridine, trigonelline, and vicine	Investigate the spatial distributions of these alkaloids in different tissues of the fresh fruit of *a. Catechu* at three different developmental stages	LC (Shim-pack UFLC Shimadzu CBM30A system) with MS/MS (Applied Biosystems Sciex QTRAP 6500 +)	Waters ACQUITY UPLC HSS T3 C18 column,1.8 µm, 2.1 × 100 mm; 0.4 mL/min; 40°C	acetonitrile with 0.04% acetic acid (A) and water with 0.04% acetic acid (B); 0–10 min,0%–95% A; 10–11 min, 95% A; 11–11.1 min 95%–5% A; 11.1–15 min, 5% A	Wu et al. (2022)
Arecoline	Develop a widely applicable and easy operation method in detection of arecoline in foods and medicines	icELISA	-	-	Wang et al. (2022b)

### 3.3 Factors influencing alkaloid content

The alkaloid content in AN is influenced by various factors, such as the variety, cultivation practices, climate, geographic location, harvesting time (growth period), processing methods, and storage. In this connection, the impact of geographic location, growth period, processing methods, and extraction techniques based on existing research are discussed.

#### 3.3.1 Geographic location

Although the AN used worldwide comes from the same species, various factors such as growing regions and weather conditions, have led to the development of different varieties of AN. As a result, there is significant variation in the alkaloid content among arecae semen varieties sold worldwide, ranging from 2 to 10 mg/g dry weight (Gupta et al., 2020). Jantarat et al. (2013) found that the content of arecoline in AN obtained from northeast Thailand ranged from 0.02% to 0.12%, whereas the content of arecoline in AN grown in China ranged from 0.22% to 0.56%. Even within the same country, regional differences in AN cultivation can have a significant impact on the content of AN metabolites. For example, Lin et al. (1992) showed that the arecoline content of AN obtained from different producing areas in China exhibited high variability. A study of AN from four regions of India (Banda Aceh, North Sumatra, West Kalimantan and West Papua) found that samples from the West Papua region had higher values than those from other regions (Sari et al., 2020). Zhang et al. (2022) investigated the metabolomics of betel quid components from four different regions in Indonesia and observed significant geographical variations in the content and concentration of metabolites. These findings underscore the importance of considering geographical and regional factors when evaluating the alkaloid content and its potential effects.

#### 3.3.2 Growth period

The content of arecoline, the most abundant alkaloid in AN, varies throughout its growth period. Research has shown that the arecoline content increases as the nut matures, reaches its maximum level, and then drastically decreases in the mature nut. When evaluating the safety and efficacy of AN consumption, this change in alkaloid content during growth is a crucial consideration. Studies have revealed that after pollination, the total alkaloid content in AN seeds initially increases and then decreases, with the highest content observed around 110 days. However, the alkaloid content in the nut’s pericarp (the outer covering) reaches its peak at around 80 days and gradually decreases as the nut continues to develop (Yan et al., 2023). This variation in alkaloid content during growth has been visualized using methods such as electrospray ionization mass spectrometry (ESI MS) and desorption electrospray ionization mass spectrometry (DESI MS) imaging. These techniques have shown that as the nut matures, white and brown regions form within the nut, and the distribution of alkaloids changes (Srimany et al., 2016). At the final stage of maturity, arecoline, arecaidine, and guvacoline tend to accumulate in the brown region of the nut, whereas guvacine is more concentrated in the white region of the nut. This segregation of alkaloids in different regions of the nut indicates that the white portion of the ripened nut may be a safer choice for consumption, as a higher proportion of alkaloids, including arecoline, is found in the brown region. Separating the white and brown regions of ripened AN can potentially allow for the safer utilization of its medicinal properties while reducing exposure to the higher alkaloid content in the brown region.

#### 3.3.3 Procession method

The processing of fresh AN can considerably affect the alkaloid content. Studies have shown variations in alkaloid levels based on different processing methods. Here are some examples: Dutta et al. (2017) examined various AN preparations consumed in India, including raw, boiled, and roasted varieties. The study found that raw AN contained the highest arecoline concentration, while boiled AN had the least, with roasted varieties exhibiting an intermediate level. In traditional Chinese medicine, AN is also processed in different ways, such as raw (fresh nuts boiled, peeled, and dried) or charred. These different processing methods can affect the content of various alkaloids. Hou et al. (2023) found that AN had the highest content of arecoline, followed by dehydroarecoline, arecaidine, and dehydroarecaidine. Roasted AN had a slightly lower alkaloid content, with differences noted in arecaidine and dehydroarecaidine. In summary, the arecoline content followed the order: raw AN > shade-dried slices > sun-exposed slices > roasted AN > charcoal.

#### 3.3.4 Extraction methods

The choice of solvent can have a significant impact on the efficiency of extracting bioactive compounds from natural products, including AN. Solvent polarity is one of the key factors influencing the extraction efficiency of phytochemicals (Suchinina et al., 2011; Zhu et al., 2020). In the context of AN seed extracts, researchers have found that extracts obtained using selected deep eutectic solvents have a higher total alkaloid content than other solvents. Ethyl acetate extracts have the lowest total phenolic content (Wang et al., 2021). Multivariate analysis results have confirmed that the choice of extraction solvent significantly influences the phytochemical composition and biological activities of AN extracts. This emphasizes the importance of carefully selecting the appropriate solvent to obtain the desired bioactive compounds from AN or other natural products, as different solvents can yield varying chemical profiles and extraction efficiencies. Researchers should consider the specific compounds they aim to extract and the properties of the solvent that will be most effective for their purposes.

## 4 Polyphenols

Polyphenols, being one of the two most biologically active substances, are the most abundant secondary metabolites in arecae semen, with an approximate 31% total polyphenol content. At least 30 polyphenol compounds have been isolated from AN (Celimuge and Xu, 2023).

### 4.1 Pharmacological activities of polyphenols

Reportedly, polyphenols exhibit various beneficial effects, including antidepressant, antihypertensive, antioxidant, anti-inflammatory, and antibacterial activities.

#### 4.1.1 Antidepressant effect

He et al. (2013) examined the antidepressant activity of total polyphenols from AN in a mouse model of depression. The results suggest that the total polyphenol extract from AN may exert a significant antidepressant effect, possibly by influencing the concentrations of monoamine neurotransmitters in brain tissue. Zhou et al. (2020) indicated that the gut microbiota could enhance the bioavailability of polyphenols by breaking them down into metabolites with higher activity and better absorption. This, in turn, can improve depression through the microbiota–gut–brain axis.

#### 4.1.2 Anti-inflammatory and antiallergic activities

AN contains a substantial amount of proanthocyanidins. Huang et al. (2010) discovered its anti-inflammatory properties and showed that can inhibit edematous inflammation induced by carrageenan and the formation of prostaglandin E2. Proanthocyanidins from AN also have effective anti-allergic properties, reducing food allergy reactions. The administration of polyphenols isolated from AN (0.05% and 0.1%, w/w) can decrease ovalbumin-induced allergic responses, including diarrhea and infiltration and degranulation of mast cells in the duodenum, as well as reduce serum ovalbumin-specific IgE and interleukin-4 levels in the duodenum (Wang et al., 2013).

#### 4.1.3 Hypotensive and antiplatelet activities

Catechins found in AN also exhibit anti-hypertensive effects. Gilani et al. (2006) found that catechins not only dilate blood vessels but also possess antiplatelet activity. In a study by Ghayur et al. (2011), catechins isolated from AN demonstrated a clear antiplatelet effect, with IC50 values of 3.630, 1.876, 2.551, 1.541, and 2.640 mg/mL for platelet aggregation induced by arachidonic acid, adenosine diphosphate, platelet-activating factor, epinephrine, and calcium ionophore, respectively.

#### 4.1.4 Antioxidant activity

Polyphenols possess antioxidant, anti-inflammatory, and antibacterial properties. Recent research has indicated that they provide protection against oxidative stress induced by hypoxia by scavenging excess free radicals and improving the blood gas parameters of hypoxic organisms (Ma et al., 2022a). The proanthocyanidins in AN, as well as flavonoids, are effective components in exerting antioxidant effects. The antioxidant capacity is related to the total polyphenol and total flavonoid content (Zhang et al., 2015). In 2022, Chaikhong et al. (2022) found that arecoline ethanol extracts, which are rich in phenolic content, demonstrated potent free radical scavenging activity. Additionally, Ma et al. (2022a) showed that arecoline acetone extract, containing a high concentration of proanthocyanidins, exhibited a significant downregulation of TPA-induced cyclooxygenase-2 expression, inhibited UVB-induced photo-damage, and mitigated premature skin aging induced by solar ultraviolet radiation.

#### 4.1.5 Antibacterial activity

HPLC analysis has shown that the major components in the extract are catechins and quercetin. Therefore, the phenolic compounds in the extract exhibit immunomodulatory activity against *Staphylococcus aureus* infection (Sari et al., 2020). The polyphenols in the ethanol extract, including catechins, epicatechin, and epicatechin gallate, possess infection-resistance activity against *Mycobacterium tuberculosis*, Gram-positive *S. aureus*, and Gram-negative *Escherichia coli* (Raju et al., 2021). Catechins also display significant antifungal properties against anthracnose fungi (Yenjit et al., 2010).

Furthermore, the acetone extracts of AN, which are rich in procyanidins, significantly inhibit cyclic adenosine monophosphate/dexamethasone-induced gluconeogenesis in mouse primary hepatocytes (Huang et al., 2013). The pro-apoptotic effect of the polyphenol-enriched AN extract on splenic lymphocytes was examined. The results indicate that the polyphenol-enriched AN extract markedly induces lymphocyte apoptosis, and the primary active constituents might be oligomeric procyanidins (Wang et al., 2009).

### 4.2 The detection criteria and methods for polyphenolic in AN

As Table 4 shows, the determination of polyphenolic compound content, primarily focusing on total polyphenols, is commonly performed using UV spectrophotometry. Wang et al. (2011) employed HPLC to determine the presence of nine polyphenolic compounds in AN fruit and seed. These compounds included gallic acid, ellagic acid, epicatechin, epicatechin gallate, catechin, chlorogenic acid, epicatechin gallate ester of ellagic acid, epicatechin, and epicatechin ester of ellagic acid. All nine components were detected in the fruit, while epicatechin, chlorogenic acid, and epicatechin ester of ellagic acid were not detected in the seeds. Among the polyphenols in the seeds, epicatechin was the most abundant (1,610 mg/kg), followed by gallic acid (622 mg/kg). Xing et al. (2019) employed ellagic acid as a reference standard and Folin reagent as the colorimetric reagent to measure the total polyphenolic content in AN using spectrophotometry. Similarly, Wang et al. (2022) employed ellagic acid as a reference standard and used the Folin colorimetric method to determine the total polyphenolic content in AN, comparing the polyphenol yield under different extraction conditions to optimize the polyphenol extraction process. Ma et al. (2022b) employed UV spectrophotometry using ferrous tartrate as a colorimetric reagent and gallic acid as the control standard to measure the total polyphenolic content in AN extracts. Additionally, to determine the content of catechin, epicatechin and protocatechuic acid in AN polyphenolic extracts, they established an HPLC analytical method. These methods aim to provide experimental evidence for the quality control and standardization of AN polyphenolic extracts. Song et al. (2022) used UHPLC-MS/MS technology to analyze and partially quantify flavonoid components in AN extracts. Their findings revealed variations in the types and quantities of flavonoid compounds among different AN parts. Hesperidin and quercetin were the predominant flavonoid components in the husk extract, whereas the seed extract contained higher levels of flavonoid compounds, including epicatechin, procyanidin B1, procyanidin B2, and L-epicatechin. Furthermore, the seed extract exhibited the highest observed antioxidant activity, the most potent inhibition of α-amylase and α-glucosidase enzymes, and the ability to inhibit angiotensin-converting enzymes relative to other extracts.

**TABLE 4:** Detection of polyphenols.

Test substance	Aim	Test method	References
Gallic acid, ellagic acid, epicatechin, epicatechin gallate, catechin, chlorogenic acid, epicatechin gallate ester of ellagic acid, epicatechin, and epicatechin ester of ellagic acid	Determine the presence of nine polyphenolic compounds in AN fruits and seeds	HPLC	Wang et al. (2011)
Total polyphenolic content	Determination of total polyphenols in areca extract	UV-Vis (Folin reagent)	Xing et al. (2019)
Total polyphenolic content	Optimize the polyphenol extraction process	UV-Vis (Folin reagent)	Wang et al. (2022a)
Total polyphenolic content and catechin, epicatechin and protocatechuic acid	Provide experimental evidence for quality control and standardization of AN polyphenolic extracts	UV-Vis (Folin reagent) and HPLC	Ma et al. (2022b)
Flavonoid components	Revealed variations in the types and quantities of flavonoid compounds among different parts of AN	UHPLC-MS/MS	Song et al. (2022)

These research methodologies are indispensable for understanding the polyphenolic content in AN, in addition to their biological activities and potential medicinal effects. Variations in components and concentrations can significantly impact the therapeutic properties of AN. By applying these methods, researchers can obtain a better understanding of the polyphenolic compounds in AN and provide data support for the quality control of related products in academic research.

### 4.3 Factors influencing polyphenolic content

Based on existing research, this section mainly introduces the effects of geographic location, growth period, and extraction methods on the polyphenol content of AN.

#### 4.3.1 Geographic location

In a study by Sari et al. (2020), AN samples from four different regions, namely, Banda Aceh, North Sumatra, West Kalimantan, and West Papua, were selected to measure the polyphenolic content in AN husks. The findings indicated that there were no significant differences in polyphenolic content among the husks from Banda Aceh, North Sumatra, and West Kalimantan. The West Papua region exhibited a significantly higher polyphenolic content in husks than the other three regions. Although all four regions share a tropical rainforest climate, they differ significantly in terms of altitude and latitude. Remarkably, West Papua, with the highest altitude and being situated in the southern hemisphere, may be the primary factor contributing to the variations in AN polyphenolic content among these regions. Correspondingly, a study of the polyphenol content of different parts of the elderberry from different altitudes and locations confirmed this conclusion (Senica et al., 2017).

#### 4.3.2 Growth period

Similar to the alkaloids, the polyphenol content in AN also changed with the growth period. Wang et al. (1997) showed that polyphenol content gradually increased with an increase in maturity. However, in another experiment, immature seeds showed a higher polyphenol content than mature seeds (Hamsar et al., 2011). Additionally, in a study by Yan et al. (2022), metabolomics analysis using gas chromatography-mass spectrometry (GC-MS) was conducted to determine the metabolites in AN husks and seeds at different developmental stages. The total flavonoid content in AN seeds was significantly higher than that in the fruit husk, with seeds reaching a maximum of 47.92% and husk reaching a maximum of 0.83%. In AN, the total flavonoid content in the seeds exhibited an increasing trend followed by a decrease, reaching its peak at 110 days of development. However, the husk showed a decreasing trend followed by an increase, with the highest content at 140 days. These studies collectively suggest that as the maturation period progresses, the polyphenolic content in AN initially increases and then gradually decreases. This result may be because different researchers have different judgments of AN maturity.

#### 4.3.3 Extraction methods

There are various methods for extracting polyphenols from AN, with the most commonly used methods being organic solvent extraction and ultrasound-assisted extraction. Fan et al. (2022) used supercritical fluid extraction to extract phenolic compounds from dried AN husks and seeds. They optimized the conditions for ultrasound-microwave synergistic extraction (UMSE). Compared with traditional liquid-solid extraction (CLSE), UMSE is a non-traditional method for the solid-phase extraction of phenolic compounds, and it offers advantages such as short reaction times, increased efficiency, and reduced energy consumption compared to CLSE. Experimental results showed that UMSE significantly improved the total phenolic content, purity, antibacterial activity, and antioxidant activity of phenolic compounds compared to CLSE (Han, 2010).

## 5 Other metabolites

Substances identified from AN extraction also include triterpenes, steroids, fatty acids, physcion, chrysophanol, and epoxyconiferyl alcohol in Table 1. (Peng et al., 2015; Liu and Chang, 2023). Terpenoids have pharmacological effects, such as antipyretic, analgesic, anti-inflammatory, antibacterial, antiviral, and anticancer effects. Steroids regulate lipid metabolism, and fatty acids are key anti-inflammatory mediators. These ingredients, together with alkaloids and polyphenols, affect the life activities of the human body, treat diseases, and threaten health. Since the content of these is not high and they are not the main pharmacologically active substance, there is almost no research on these ingredients in AN.

## 6 Omic studies in AN

Currently, in quality and safety research on AN, the technologies applied extend beyond the evaluation of biological activity and the determination of major pharmacological components. They also encompass genomic, metabolomic, and transcriptomic techniques.

### 6.1 Omics and *A. catechu* germplasm

The genome of AN has recently been published. Yang et al. (2021) presented a chromosome-scale reference genome assembly with 92.92% of the genes functionally annotated. This annotation included genes responsible for the biosynthesis of flavonoids, anthocyanins, monoterpenoids, and their derivatives, indicating an enrichment and expansion of gene families. However, despite the recognition of 40–50 *Areca* species, the geographic origins of AN, and its subsequent diffusion and diversification remain inadequately documented. A gene-typing method targeting 34 chloroplast DNA microsatellites was developed to unravel the historical aspects of AN evolution (Raimondeau et al., 2021). This method can be used to trace maternal lineage propagation, proving effective in the analysis of plant specimens. It is essential to elucidate the regulatory mechanisms governing fruit shape in the pursuit of cultivating superior AN varieties. Ding et al. (2023) categorized fruit from 137 AN germplasm resources into three types (spherical, elliptical, and cylindrical) based on the fruit shape index. They identified 86 candidate genes associated with fruit morphology. These candidate genes encode proteins, such as UDP-glucose transferase 85A2, ABA-responsive element-binding factor GBF4, E3 ubiquitin-protein ligase SIAH1, and leucine-rich repeat receptor-like serine/threonine-protein kinase ERECTA. Furthermore, in *A. catechu*, the frequent occurrence of small fruit detachments leading to significant yield losses has been observed. Researchers found that the genes encoding plant-specific DNA binding with one finger (DOF) transcription factors exhibited a uniform upregulation in the abscission zone, suggesting the potential role of DOF transcription factors in the regulation of fruitlet abscission in *A. catechu* (Li et al., 2022).

### 6.2 Omics and metabolites in AN

Non-targeted metabolomics methods have been employed to identify new compounds with potential medicinal and physiological activities in AN (Wu et al., 2021). The metabolic profile of Indonesian betel quid was analyzed using non-targeted GC-MS, and 92 plant chemical substances with the exception of alkaloids, primarily including benzene ring compounds, terpenes, acids, aldehydes, alcohols, and esters, were identified (Zhang et al., 2022). Lai et al. (2023) utilized non-targeted metabolomics to identify 331 metabolites from the roots, stems, and leaves of *A. catechu*, including 107 flavonoids, 71 lipids, 44 amino acids and derivatives, and 33 alkaloids. Thirty-six genes were identified using combined transcriptome and metabolomic analysis examining the biosynthetic processes underlying metabolic variations in *A. catechu* tissues. Flavonoid biosynthesis was controlled by transcription factors AcMYB5 and AcMYB194. Zhou et al. (2023) conducted a comprehensive analysis of the mechanisms underlying the accumulation of B vitamins during the development of AN through a combination of transcriptomics and targeted metabolomics. A total of 88 structural genes related to B vitamin biosynthesis were identified. The study obtained the metabolic profiles of 6 B vitamins at different AN developmental stages. The key transcription factors responsible for regulating the accumulation of thiamine and riboflavin in AN were identified, including AcbZIP21, AcMYB84, and AcARF32.

### 6.3 Omics and bioactivity of AN

A comprehensive approach integrating metabolomics and network pharmacology was employed by Li et al. (2023) to elucidate the potential mechanisms underlying AN addiction. Network pharmacology analysis revealed that all crucial targets associated with AN addiction were modulated by arecoline, highlighting the significance of the G protein-coupled receptor signaling pathway. Furthermore, analysis of plasma and fecal metabolomes following arecoline intervention in mice suggested that this component may affect the dopamine and 5-hydroxytryptamine systems by modulating the biosynthesis of phenylalanine, tyrosine, and tryptophan, as well as the metabolism of phenylalanine, primary bile acids, glycerophospholipids, and the structure of the intestinal microbiota.

In a serum metabolomics study examining the toxicity of arecae semen and its underlying mechanisms, arecae semen demonstrated distinct cardiotoxic effects and hindered normal growth in Wistar male rats (Lin et al., 2020). The differentiation in metabolic profiles revealed 19 metabolites identified as potential biomarkers in rats treated with arecae semen.

Using ultra-high-performance liquid chromatography-time-of-flight mass spectrometry (UHPLC-TOF-MS) analysis of urine in mice, a metabolomics approach was employed to study the metabolism of arecoline and arecaidine (Giri et al., 2006). Eleven arecoline metabolites were identified, including arecaidine, arecoline N-oxide, arecaidine N-oxide, N-methylnipecotic acid, N-methylnipecotylglycine, arecaidinylglycine, arecaidinylglycerol, arecaidine mercapturic acid, arecoline mercapturic acid, and arecoline N-oxide mercapturic acid, as well as nine unidentified metabolites. The predominant metabolite for both arecoline and arecaidine was N-methylnipecotic acid. An additional noteworthy metabolite detected was the monoacylglyceride of arecaidine.

A previous study on AN chewing and OSCC suggested that exposure to carcinogenic substances present in AN may lead to genomic instability detected through oral tumor subsets by SSR PCR (Zienolddiny et al., 2004). Later research on the mutational characteristics of AN chewing-related tongue cancer confirmed that in the general population, AN chewing-related tongue cancer exhibits distinct mutational features compared to non-AN chewing-related tongue cancer. These features are associated with frequent mutations in the RASA1 gene and CpG islands throughout the entire genome (Zhang et al., 2019). Another study investigated the impact of AN chewing on salivary proteomics through MS (Sultan et al., 2020). MALDI-MSI was employed to generate a profile of peptides in AN consumers and a control group. Thirteen peptide peaks were significantly altered (*p* < 0.05) in AN addicts compared to the control group. These significant peptides corresponded to proteins such as cystatin SN, cystatin S, α2 macroglobulin, complement C3, apolipoprotein E, serum albumin, matrix metalloproteinase-9 deleted in malignant brain tumor protein 1, zinc-alpha-2-glycoprotein, and protein S100A8. Most of these proteins interact with each other, and some serve as biomarkers for malignant oral tumors.

The intestinal microbiota exhibits a significant response to arecoline intake. Based on shotgun metagenomic sequencing using a metagenomic shotgun approach, Xu et al. (2023a) investigated the impact of arecoline on intestinal microbiota. The results indicated that arecoline promoted lipid metabolism in mice, manifested by a significant reduction in serum total cholesterol, triglycerides, and hepatic total cholesterol levels, leading to a decrease in abdominal fat accumulation. Arecoline intake significantly modulates the levels of the neurotransmitters serotonin and norepinephrine in the brain. Notably, arecoline intervention markedly increases serum interleukin-6 and lipopolysaccharide levels, inducing inflammation in the body. Arecoline significantly reduces hepatic glutathione levels and increases malondialdehyde levels at a high dose, causing hepatic oxidative stress. Arecoline intake leads to intestinal damage by stimulating the release of IL-6 and IL-1β. In another study, arecoline demonstrated an ameliorative effect on intestinal damage induced by constipation (Xu et al., 2023b). By measuring symptoms related to constipation, intestinal microbiota, short-chain fatty acid content in the cecum, and gene expression in the colon, the impact of arecoline on constipation was explored. Arecoline intervention significantly downregulated gene expression associated with intestinal diseases, alleviating constipation induced by loperamide. Furthermore, an experiment on non-alcoholic fatty liver disease rats revealed through analysis of intestinal metabolomics and 16S rRNA sequencing that arecoline has lipid-lowering effects (Zhu et al., 2023). This effect may be mediated through intestinal metabolites, gut microbiota, and the Butyricicoccus/Christensenella/Coriobacteriaceae-COX2/PGE2 pathway.

## 7 Discussion and conclusion

### 7.1 Clinical studies have shown that areca nut has no obvious side effects

The mainstream view is that chewing AN causes oral cancer, and the alkaloid components in AN play a key role. Although alkaloids found in AN are associated with various toxic effects, in clinical practice, traditional Chinese physicians prescribe AN-based treatments based on traditional Chinese medicine theory and individual patient conditions. AN is often processed through decoction (boiling in water) before being administered to patients, and the dosages used are significantly lower than those consumed when chewing AN. Investigations reveal that there have been no reported cases of severe adverse reactions associated with the use of AN-containing Chinese herbal medicines, such as “Simo Decoction” (Sun et al., 2017). However, it is essential for patients to follow the guidance of trained healthcare professionals and exercise caution when using any herbal treatment, including those containing AN, as individual reactions may vary.

### 7.2 Alkaloids are the primary components in AN responsible for addiction and carcinogenic effects

In AN, the four primary alkaloids are arecoline, arecaidine, guvacine, and guvacoline, with arecoline having the highest concentration. Arecoline is the predominant and leading inducer of addiction and carcinogenic effects. Consequently, researchers and experts have directed significant attention to understanding the content and biological effects of arecoline in AN. Generally, arecaidine, guvacine, and guvacoline have lower concentrations than arecoline; however, these proportions are not absolute. The relative content of these four alkaloids varies based on factors such as AN variety, geographical location, degree of ripeness, and processing methods. Therefore, it is not entirely reasonable to focus exclusively on studying the content and biological effects of arecoline. The lower concentrations of arecaidine, guvacine, and guvacoline should not be disregarded. In research, comprehensive assessments should include all four alkaloids to accurately reflect the safety and efficacy of AN. Given these findings, for diverse purposes, users may select AN of different varieties, growth periods, and origins and use various processing methods to either reduce or increase specific components.

### 7.3 Alkaloids and polyphenols: harnessing beneficial pharmacological effects

AN has been used in China since the Western Han dynasty, as documented in ancient Chinese medical texts. These texts record various medicinal combinations involving AN, demonstrating its efficacy in treating conditions such as oppressive sensations in the chest, heartache, lower back pain, urinary and bowel irregularities, intestinal parasitism, athlete’s foot, and oral ulcers. Subsequent scholarly investigations into the pharmacological properties of AN extracts have revealed their antidepressant, antimicrobial, anti-aging, insecticidal, anti-osteoporotic, hepatoprotective, and anti-inflammatory effects (Dar et al., 1997; Lee and Choi, 1999; Pithayanukul et al., 2009; Li et al., 2017; Jam et al., 2021; Sharaf et al., 2021; Kweon et al., 2022; Puyathorn et al., 2022). In a recent study, extracts from arecae semen demonstrated inhibitory effects on snake venom (More et al., 2022). Summarizing modern pharmacological experiments on AN alkaloids and polyphenols has revealed their diverse biological functions, including insecticidal, anti-inflammatory, antimicrobial, hypotensive, anxiolytic, antidepressant, gastrointestinal improvement, and antioxidant properties. Alkaloids and polyphenols are the primary substances responsible for the therapeutic effects of AN. Although other components in AN also possess a variety of biological functions, their lower concentrations relegate them to secondary roles. Thus, they have not garnered as much attention from researchers. Furthermore, the therapeutic effects or toxicity of AN vary depending on the solvent and extraction methods employed. This variation is ascribed to alterations in the content, proportion, and types of different components in the extracts resulting from these processing methods. The question of how to optimize processing to maximize efficacy while minimizing side effects remains a subject that requires further exploration.

### 7.4 Integrated multi-omics research has become mainstream

Presently, omics studies have become the primary means for discovering active compounds in AN and for understanding the pathways and mechanisms of AN metabolite synthesis and accumulation. They are also applied to tracing the historical spread of AN and cultivating superior varieties. Concurrently, integrated multi-omics research plays a crucial role in enhancing our understanding of AN metabolism in the body and its impact on the internal microenvironment.

The discovery of potential medicinal and physiologically active compounds in AN using metabolomics is just the beginning. It is crucial to employ network pharmacology to identify potential targets, conduct structure–activity relationship studies, isolate and identify components, and subsequently proceed with efficacy research, which will help to explore and develop the value of AN. Additionally, future research can leverage omics technologies for comprehensive comparative studies on AN from different regions of the world and growth stages. Based on this foundation, researchers can optimize the use of different AN varieties or engage in breeding efforts to meet diverse needs. Simultaneously, it is crucial to define the optimal harvesting period for AN. Studying the changes in metabolites or pharmacological effects during the maturation process across different production areas can guide the selection of suitable harvesting times for specific purposes. Further, establishing industry-wide consensus or standards for harvest times is essential. For AN used in traditional Chinese medicine, systematic research should be conducted to support regulations regarding harvesting times and processing methods.

Researchers need to be mindful that the medicinal and chewing forms of AN consumption are not the same when using omics technologies to study the potential effects of AN on the human body. Research conducted on the correct form of use can more accurately reflect its impact on the human body. Additionally, whether consumed as food or for medicinal purposes, AN is used in its entirety. Therefore, studying individual components cannot be entirely rational for representing the holistic nature of AN.

### 7.5 AN quality detection

Quality is the primary factor influencing the value of products and people’s choices regardless of whether it is used for consumption or medicinal purposes. Quality can be affected by factors such as the growing environment, variety, processing methods, and extraction techniques. Currently, the presence and content of medicinal constituents are key indicators for evaluating their quality. However, unlike chemical pharmaceuticals with simpler compositions, traditional Chinese medicine contains a multitude of components and medicinal constituents, making it challenging for researchers to determine the criteria for assessing the quality of herbal medicines.

Alkaloids and tannin concentrates are considered the characteristic components of AN, and alkaloids and phenolic substances are the main bioactive components. Alkaloids have received the most attention in research on *A. catechu*, with arecoline accounting for the vast majority and other alkaloids being investigated rather infrequently. Polyphenolic flavonoids, primarily total polyphenol and other components of polyphenols, have also received attention. There is a significant change in the content and types of polyphenols in different parts of *A. catechu*. Schaftoside and diosmetin are the most abundant in husks, and catechins, followed by tannic acid, are the most abundant in seeds (Song et al., 2022). Therefore, arecoline, arecaidine, guvacine, guvacoline, and catechin are potential quality markers and can be used as index components to evaluate the quality of arecae semen. In addition, the detection of a single substance is always limited in representativeness when considering the multi-component and multi-target characteristics of traditional Chinese medicine. However, too many components will make the detection work time consuming and laborious. A simpler and more comprehensive method of AN quality determination needs to be explored.

## Abbreviations

OSF, oral submucous fibrosis; OSCC, oral squamous cell carcinoma; OPSCC, oropharyngeal squamous cell carcinoma; MIC, minimal inhibitory concentration; PI3K/AKT, phosphatidylinositol 3-kinase/protein kinase B; PI3K/AKT/mTOR, phosphatidylinositol 3-kinase/protein kinase B/mammalian target of rapamycin; HPLC, high-performance liquid chromatography; UV, ultraviolet; UHPLC-MS/MS, ultra-high-performance liquid chromatography-tandem mass spectrometry; IMS, ion mobility spectrometry; MALDI-MSI, matrix-assisted laser desorption/ionization mass imaging spectrometry; IC50, half maximal inhibitory concentration; ESI, electrospray ionization; DESI, desorption electrospray ionization; GC-MS, gas chromatography-mass spectrometry DOF, DNA-binding with one finger; SSR PCR. simple sequence repeats polymerase chain reaction.
